# Usefulness of Serum IgG4 and Thyroid Stimulating Hormone Levels in Differentiating Headache

**DOI:** 10.31662/jmaj.2021-0220

**Published:** 2022-06-17

**Authors:** Yoshiyuki Nakatsuji, Shinya Fukuzawa, Isao Arima, Kaori Yamamoto

**Affiliations:** 1 Department of Gastroenterology and Internal Medicine, Marunouchi Hospital, Matsumoto, Japan; 2 Department of Gastroenterology, Marunouchi Hospital, Matsumoto, Japan

**Keywords:** IgG4-related disease, Hypophysitis, Cholecystitis, Thyroid stimulating hormone, Headache, Prednisolone

A 67-year-old man was admitted with a headache. He had a history of cholecystectomy for immunoglobulin G4 (IgG4)-related cholecystitis and hypophysitis, diagnosed 7 and 3 years ago, respectively. Moreover, a year ago, he underwent surgery for unruptured cerebral aneurysm. Hypophysitis diagnosis was based on pituitary enlargement observed on head contrast-enhanced computed tomography (Figure 1A and B), IgG4-positive cells in the gallbladder wall at cholecystectomy (Figure 2A and B), and high serum IgG4 levels (883 mg/dL) ^(1), (2)^. It was accompanied by headache and hypopituitarism, a substantial decrease in the thyroid stimulating hormone (TSH) level, which improved to normal levels following the administration of 30 mg/day prednisolone ^(3)^. Headache was present during cerebral aneurysm surgery (Figure 3: yellow dot); however, IgG4 and TSH levels remained stable. The aforementioned levels in X + 7 years were 206 mg/dL and 0.025 μIU/mL, respectively, which helped differentiating his headache and increasing the dosage of prednisolone (Figure 4) ^(4)^.

**Figure 1.**
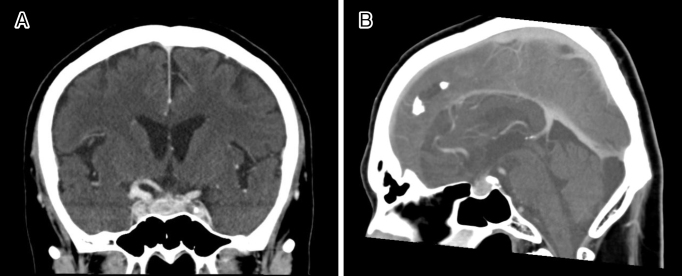
A. Contrast-enhanced coronal computed tomography (CT) of the head displaying an enlarged pituitary gland and stalk. An aneurysm of the right internal carotid artery is suspected. B. Contrast-enhanced sagittal CT of the head displaying an enlargement of the pituitary gland and stalk.

**Figure 2.**
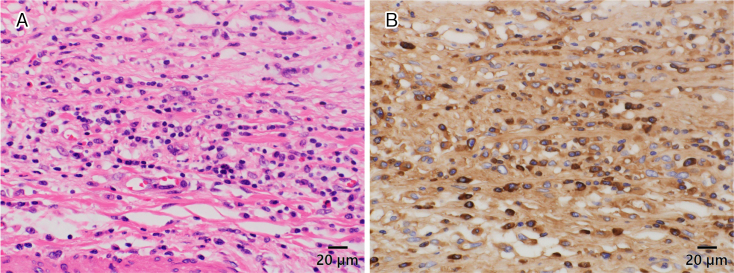
A. Hematoxylin-eosin staining of the gallbladder wall. Collagen fibers are stained pink and display growth. Significant infiltration of the lymphoplasmacytic cells. B. Immunoglobulin G4 (IgG4)-positive immunohistochemical image of the gallbladder wall. Infiltration of IgG4-positive plasma cells (stained brown) is observed. More than 40% of IgG-positive plasma cells were IgG4-positive, and these cells are >10 cells/high-power field.

**Figure 3.**
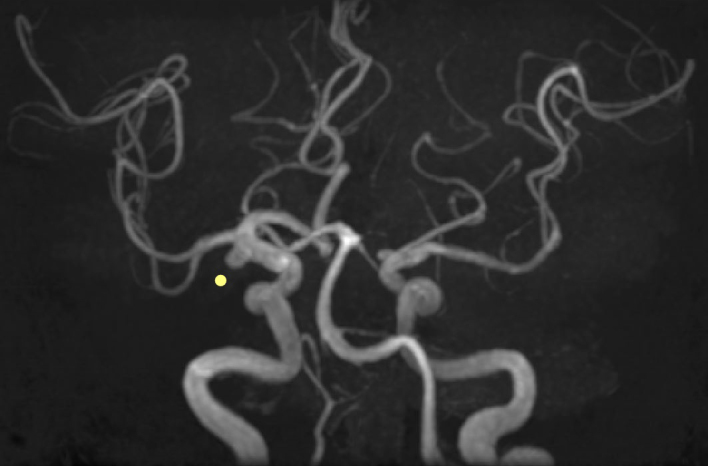
Magnetic resonance angiography of the head displaying an aneurysm (yellow dot) of the right internal carotid artery.

**Figure 4.**
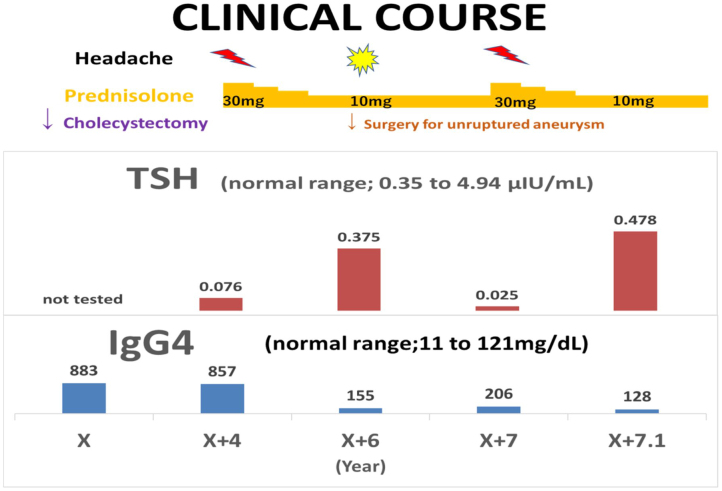
Clinical course of the patient. 25 μg/day of levothyroxin administration has been started since X + 4 years. The serum adrenocorticotropic hormone (ACTH) level was less than 2.0 pg/mL (N: 7.2-63.3) and the serum growth hormone (GH) level was 0.34 ng/mL (N: ≥2.47). In addition, the free T4 (FT4) level was 0.69 ng/dL (N: 0.7-1.7). A comparison of head computed tomography scans at X + 4 and X + 6 years suggested that prednisolone reduced the approximate pituitary volume by approximately one-half (1563 vs. 839 mm^3^).

## Article Information

### Conflicts of Interest

None

### Acknowledgement

We thank Maki Miyata M.D. and Masahiro Agata M.D. at Ichinose Neurosurgical Hospital in Matsumoto for the neurosurgical operation of cerebral aneurysm. We also thank Jyun Igarashi M.D. at Department of Surgery of Marunouchi Hospital for performing cholecystectomy, Tsuyoshi Tada M.D. at Department of Neurosurgery of Marunouchi Hospital for his helpful discussion, and Masayoshi Hayama Ph.D. at Department of Clinical Pathology of Marunouchi Hospital for his fantastic work of histopathology. There are no sources of financial support.

Our manuscript has been edited by a native English speaker of editage^Ⓡ^.

### Author Contributions

YN planned and prepared this manuscript; SF and IA provided their support for preparing the article; KY provided support for submitting the tissue samples and helped finalizing the article.

### Approval by Institutional Review Board (IRB)

This study did not require IRB approval.

### Patient Consent

Informed consent was obtained from patient to publish this case, including pictures.
